# Barriers to Prevention and Treatment of Type 2 Diabetes Mellitus Among Outpatients in Belarus

**DOI:** 10.3389/fcdhc.2021.797857

**Published:** 2022-01-06

**Authors:** Anastasiya Sachkouskaya, Tamara Sharshakova, Dmitry Kovalevsky, Maria Rusalenko, Irina Savasteeva, Aya Goto, Hirohide Yokokawa, Atsushi Kumagai, Jumpei Takahashi

**Affiliations:** ^1^ Public Health Department and Public Health Services, Gomel State Medical University, Gomel, Belarus; ^2^ Administration Department, Republican Research Center for Radiation Medicine and Human Ecology, Gomel, Belarus; ^3^ Information and Analytical Department, Republican Research Center for Radiation Medicine and Human Ecology, Gomel, Belarus; ^4^ Center for Integrated Science and Humanities, Fukushima Medical University, Fukushima, Japan; ^5^ Department of General Medicine, Juntendo University, Tokyo, Japan; ^6^ Department of Radiation Emergency Medicine, The National Institutes for Quantum and Radiological Science and Technology, Chiba, Japan; ^7^ Office of Global Relations, Nagasaki University, Nagasaki, Japan

**Keywords:** diabetes mellitus, behavioral risk factors, treatment adherence, prevention adherence, barriers

## Abstract

**Objectives:**

This study aimed to determine the main barriers and reasons for non-adherence to preventive measures and treatment for type 2 diabetes mellitus among outpatients in Belarus.

**Methods:**

An anonymous questionnaire survey was conducted with 814 adults aged 18 years and over who visited outpatient health care units and hospitals in Belarus. The questionnaire was developed to analyze the perceived barriers that limit adherence to preventive measures and treatment for type 2 diabetes.

**Results:**

The proportion of respondents who reported doing daily physical activity was 53.2%, and 46.6% consumed at least 400 grams of fruit and vegetables per day. Among the 42.8% of respondents with a prescribed treatment for type 2 diabetes mellitus, 50.1% sometimes forgot to take their medicine. The specific barriers to treatment most frequently identified by survey respondents were “Financial situation” (23.5% of respondents), and “Fear of side effects” (25.2%). Those for lifestyle instructions were “Insufficient knowledge” (29.3%), “Financial situation” (27.9%), and “Lack of motivation” (21.7%).

**Conclusions:**

The study revealed that the main barriers to adherence were insufficient knowledge of diabetes and its treatment and an underestimation of the role of behavioral risk factors in health, combined with financial difficulties. We recommend that physicians should take a patient-centered approach to raising awareness of behavioral risk factors for type 2 diabetes mellitus, and suggest that the importance of adhering to preventative measures and treatment should be promoted in consultations in Belarus.

## Introduction

The increasing prevalence of diabetes worldwide might indicate that primary prevention measures and their promotion are insufficient in the populations of most countries globally (1). There are 352 million people living with diabetes, most of whom are working adults aged 20–64 years (2). This population is expected to grow to 417 million by 2030 and to 486 million by 2045. These projections indicate that diabetes not only has a growing human impact, but also that it will place a serious and increasing strain on productivity and economic growth in the coming decades. In the Republic of Belarus, as of January 1, 2020, there were 352,538 patients with diabetes under follow-up, including type 1 diabetes mellitus (n=18,110) and children (n=2,438) (3). The reported annual increase of patients with diabetes in the country is 5%–8%.

According to a recent review by Galaviz and colleagues (1), lifestyle modification can lower diabetes risk. For example, the US Diabetes Prevention Program (DPP) (4), the Finnish Diabetes Prevention Study (5), and the Da Qing Diabetes Prevention Study (6) have demonstrated that lifestyle/behavioral therapy featuring an individualized reduced-calorie meal plan is highly effective in preventing type 2 diabetes mellitus and improving other cardiometabolic markers, such as blood pressure, lipids, and inflammation. The DPP also found that intensive lifestyle intervention could reduce the risk of incident type 2 diabetes mellitus by 58% over 3 years (7). With such solid evidence, more attention needs to be paid on the delivery of the interventions (1).

An important measure for reducing the burden of diabetes is the participation of patients in the management of the disease. The American Diabetes Association highlights the importance of patient-centered care, which is defined as care that considers individual patient comorbidities and prognoses; is respectful of and responsive to patient preferences, needs, and values; and ensures that patient values guide all clinical decisions (8). Awareness of the risk factors, symptoms, and complications of type 2 diabetes mellitus in the general population has an important impact on people’s perceptions of this disease and its primary prevention. An important goal is not only to increase awareness of risk factors for diabetes among the general population, but also to improve adherence to preventive measures and treatment. At the national level, individuals’ non-adherence affects the health care system through increased morbidity and mortality and reduced quality of life, which in turn leads to rising health care costs (9–12).

The World Health Organization defines adherence as “the extent to which the patient follows medical instructions” (13). The most difficult challenge is achieving high adherence to therapy for chronic disorders, such as diabetes, which requires lifelong medication, compliance, and regular medical consultations. For effective type 2 diabetes management, it is important to achieve adherence not only to medication, but also to lifestyle instructions, such as increasing physical activity, specific dietary regimens, smoking cessation, and strict monitoring of blood glucose levels (9, 10, 14). The best way to achieve good medical and health behavioral adherence is to identify and assess the factors that lead to the non-adherent behavior, so that interventions can be designed to minimize or remove these factors. As previous studies have shown, there are various interwoven factors underpinning diabetes self-management (15). Brundisini and colleagues’ qualitative meta-synthesis identified seven categories of patients’ experiences of adherence: emotional experiences as positive and negative motivators to adherence, intentional non-compliance, patient-provider relationship and communication, information and knowledge, medication administration, social and cultural beliefs, and financial issues (9). However, in the Republic of Belarus, such patients’ perspectives of barriers to prevention and treatment of type 2 diabetes mellitus remain poorly studied (16). Therefore, our study aimed to determine the main real-life barriers to adherence in the prevention and treatment of type 2 diabetes mellitus among outpatients in Belarus.

## Methods

This was a cross-sectional study. Data were collected between January and April 2020 from the population who applied for medical assistance at outpatient health care units (organizations that provide primary health care) and hospitals in Gomel, Belarus. Namely, the survey sites were the Republican Research Center for Radiation Medicine and Human Ecology in Gomel, Gomel Regional Clinical Polyclinic, Gomel Central Polyclinic (subsidiary No. 8), and Chechersk Central District Hospital. These institutions are where patients have first contact with doctors. The main inclusion criteria were patients with a diagnosis of diabetes and/or with risk factors for diabetes [such as family history of diabetes, overweight (body mass index of 25.0 or over) or obesity (body mass index of 30.0 or over), and physical activity less than 3 times a week]. The main exclusion criteria were medical workers and patients with acute conditions, exacerbations of chronic diseases, and mental disorders, as well as patients with terminal illness. All patients meeting the above criteria during the study period were recruited.

An anonymous questionnaire was used as the investigation method. The respondents’ answers were recorded on a questionnaire sheet by a researcher during an interview or by the respondents themselves. The questionnaire included the following items: demographic information (sex, age, and education), lifestyle items (at least 30 minutes of physical activity daily, three times a week, less than three times a week, or none at all), smoking, drinking (never, once a month or less, 2–4 times a month, 2–3 times a week or more), diet (consumption of 400 grams of fruit and vegetables per day), and barriers that impede adherence to a healthy lifestyle and treatment regimen.

Statistical analyses were carried out using Microsoft Office Excel 2013 (Microsoft Corp.; Redmond, WA, USA) and Portable Statistica 8 (StatSoft, Inc.; Tulsa, OK, USA).

The study was approved by the General Directorate of Health Services, Gomel. All respondents provided informed written consent prior to participation in the study and agreed to anonymous use of their data. Participation was voluntary.

## Results

Among 814 respondents, 72.0% (n=586) were women and 28.0% (n=228) were men. The age distribution was as follows: 18–24 years as teens and young adults, 3.7% (n=31); 25–34 years, 8.7% (n=71); 35–44 years, 18.9% (n=154); 45–59 years, 31.9% (n=260); and 60 years or older, 36.6% (n=298). As for educational attainment, the lower secondary level accounted for 2.9% (n=24); secondary level, 3.6% (n=30); basic level, 45.8% (n=178), and higher level, 47.6% (n=388). There was no statistically significant difference between groups with and without diabetes for these basic characteristics, while the presence of risk factors for diabetes mellitus was significantly higher in the group with diabetes than those without the disease. The proportion of those with a family history of diabetes was 34.9% in the diabetes group and 24.5% in the non-diabetes group, that of overweight was 23.5% and 10.7%, obesity was 29.9% and 10.3%, and physical activity less than 3 times a week was 39.2% and 27.7%, respectively.

As for daily lifestyles (**Table 1**), the proportion of respondents who reported doing daily physical activity was 53.2% (n=433), which was statistically higher among those without diabetes than those with diabetes (60.8% and 42.9%, respectively; p<0.001). In terms of alcohol consumption, 34.6% of respondents (n=282) answered never, which was higher among those with diabetes than those without (40.9% and 29.9%, respectively; p<0.001). Additionally, 46.6% of respondents (n=379) consumed at least 400 grams of fruit and vegetables per day, which was higher among those without diabetes than those with diabetes (50.5% and 41.5%, respectively; p=0.01).

**Table 1:** Lifestyle characteristics among the respondents.

Lifestyle characteristics		Total n = 814 (100%)	With DM n = 349 (42.9%)	Without DM n = 465 (57.1%)	p value
Physical activity	Daily	433 (53.2)	150 (42.9)	283 (60.8)	<0.001
	Three times a week	117 (14.4)	62 (17.8)	55 (11.8)	
	Less than three times a week	167 (20.5)	80 (22.9)	87 (18.7)	
	No	97 (11.9)	57 (16.3)	40 (8.6)	
Smoking	No	703 (86.4)	292 (83.7)	412 (88.4)	0.06
	Yes	111 (13.6)	57 (16.3)	54 (11.6)	
Alcohol consumption	Never	282 (34.6)	143 (40.9)	139 (29.9)	<0.001
	Once a month or less	389 (47.8)	142 (40.7)	247 (53.1)	
	2–4 times a month	120 (14.7)	50 (14.3)	70 (15.1)	
	2–3 times a week or more	23 (2.8)	14 (4.0)	9 (1.9)	
Consumption of 400 grams of fruit and vegetables per day	Yes	379 (46.6)	144 (41.5)	234 (50.5)	0.01
	No	435 (53.4)	204 (58.5)	229 (49.5)	

Data are shown as n (%) within “total”, “with DM”, or “without DM”, except for the proportion in the top row indicating proportions of those with and without DM.

Chi-Square test was used.

DM, diabetes mellitus.

The proportion of respondents with a prescribed treatment for type 2 diabetes mellitus was 42.8% (n=349), of whom 50.1% sometimes forgot to take their medicine, 49.6% were sometimes inattentive to hours of drug administration, 28.4% skipped medication if they felt well, and 35.5% skipped the next medication intake if they felt unwell after taking medication. Reasons given in response to “Why don’t you take a doctor-prescribed treatment?” were “Financial situation” in 23.5% of respondents, “Fear of side effects” in 25.2%, “Lack of information about benefits of drug therapy” in 13.8%, “Lack of motivation” in 18.6%, and “Other” in 26.1%. The main “Other” reason indicated was lack of time.

In response to the question, “What impedes your adherence to healthy lifestyle instructions?”, “Insufficient knowledge” was the reason given by 29.3% of the respondents, “Financial situation” by 27.9%, “Lack of motivation” by 21.7%, “Other” by 18.2%, “Lack of faith in the effectiveness of measures taken” by 12.0%, and “Low availability of medical care” by 8.1%. As shown in **Table 2**, the main reasons for low adherence to a healthy lifestyle in the 18–24 age group were “Lack of motivation” and “Financial situation”. With increasing age, a larger proportion of the respondents gave the reason “insufficient knowledge”. In almost all the age groups, “Low availability of medical care” was indicated the least.

**Table 2:** Barriers to adherence to healthy lifestyle instructions in the study respondents: comparison by age group.

Possible reasons	Total n = 814	18-24 years n = 31	25-34 years n = 71	35-44 years n = 154	45-59 years n = 260	60 years or older n = 298
Lack of knowledge	239 (29.3)	4 (12.9)	22 (31.0)	36 (23.4)	73 (28.1)	104 (34.9)
Financial situation	227 (27.9)	8 (25.8)	19 (26.8)	40 (26.0)	74 (28.5)	86 (28.9)
Lack of motivation	177 (21.7)	11 (35.5)	15 (21.1)	35 (22.7)	55 (21.2)	60 (20.1)
Other	148 (18.2)	6 (19.4)	15 (21.1)	37 (24.0)	56 (21.5)	34 (11.4)
Lack of faith in the effectiveness of measures taken	98 (12.0)	3 (9.7)	3 (4.2)	28 (18.2)	27 (10.4)	37 (12.4)
Low availability of medical care	66 (8.1)	2 (6.5)	8 (11.3)	9 (5.8)	14 (5.4)	33 (11.1)

Multiple responses permitted. Data are shown as n (%). The denominator is the total number of people in each group.

Answers were then compared by the diagnosis of diabetes, as well as gender and education (**Table 3**). Among patients with diabetes, 38.4% indicated “Financial situation” as the main reason for low adherence to a healthy lifestyle, while 28.6% of the respondents without diabetes indicated “Insufficient knowledge”. A statistically significant difference was observed for “Financial situation” (p<0.001) and “Other” (p=0.001). Further comparison by gender showed that a significantly higher proportion of women selected “Financial situation” (p=0.004), and a comparison by education showed that a higher proportion of those without a higher education selected “Financial situation” (p=0.02), “Insufficient level of knowledge” (p=0.04), and “Other” (p<0.001).

**Table 3:** Barriers to adherence to healthy lifestyle instructions in the study respondents: comparison by the diagnosis of diabetes, gender, and education.

Possible reasons	With DM n = 349	Without DM n = 465	p value	Men n=228	Women n=586	p value	Higher education n=388	Lower education n=426	p value
Financial situation	134 (38.4)	93 (20.0)	<0.001	47 (20.6)	180 (30.7)	0.004	93 (23.9)	134 (31.4)	0.02
Insufficient level of knowledge	106 (30.4)	133 (28.6)	0.59	73 (32.1)	166 (28.3)	0.30	100 (25.8)	138 (32.2)	0.04
Lack of motivation	78 (22.3)	99 (21.2)	0.73	51 (22.3)	125 (21.3)	0.78	80 (20.6)	96 (22.5)	0.55
Lack of faith in the effectiveness of measures taken	50 (14.3)	48 (10.3)	0.10	35 (15.4)	62 (10.6)	0.07	51 (13.1)	47 (11.03)	0.32
Other	46 (13.2)	102 (21.9)	0.001	39 (17.1)	109 (18.6)	0.69	97 (25)	52 (12.2)	<0.001
Low availability of medical care	23 (6.5)	43 (9.2)	0.19	23 (10.1)	43 (7.3)	0.20	32 (8.2)	34 (7.9)	0.89

Multiple responses permitted. Data are shown as n (%). The denominator is the total number of people in each group.

Fisher’s exact test was used.

DM, diabetes mellitus.

Lower education group included lower secondary, secondary, and basic levels.

We also asked the respondents, “What, in your opinion, creates the barriers to following a healthy lifestyle in the general population (in Belarus)?” (**Table 4**). The answer “Financial situation” was given by 42.9% of the respondents, “Lack of knowledge” by 42.3%, “Lack of motivation” by 18.4%, and “Psychological state” by 17.6%. Answers to this item were compared between those with and without diabetes mellitus. In those with a diagnosis of diabetes mellitus, nearly half (48.4%) answered “Financial situation”, followed by “Lack of knowledge” at 40.4%, “Psychological state” at 17.7%, and “Lack of faith in the effectiveness of the measures taken” at 16.9%. The answers most frequently indicated by the respondents without a diagnosis of diabetes mellitus was “Lack of knowledge” at 43.8%, “Financial situation” at 38.9%, “Lack of motivation” at 21.1%, and “Psychological state” at 17.4%. A statistically significant difference was observed for “Financial situation” (p=0.01) and “Lack of motivation” (p=0.03).

**Table 4:** Barriers to daily adherence to healthy lifestyle in the general population: comparison between those with and without diabetes.

Possible reasons	Total n = 814	With DM n = 349	Without DM n = 465	p value
Financial situation	350 (42.9)	169 (48.4)	181 (38.9)	0.01
Lack of knowledge	345 (42.3)	141 (40.4)	204 (43.8)	0.35
Lack of motivation	150 (18.4)	52 (14.9)	98 (21.1)	0.03
Psychological state	143 (17.6)	62 (17.7)	81 (17.4)	0.93
Lack of faith in the effectiveness of measures taken	120 (14.7)	59 (16.9)	61 (13.1)	0.14
Low availability of medical care	98 (12.0)	38 (10.9)	60 (12.9)	0.45
Mistrust in the health care system	81 (9.9)	31 (8.8)	50 (10.7)	0.41
Other	44 (5.4)	15 (4.3)	29 (6.2)	0.27

Multiple responses permitted. Data are shown as n (%). The denominator is the total number of people in each group.

Fisher’s exact test was used.

DM, diabetes mellitus.

## Discussion

Descriptive analysis of lifestyle characteristics among our respondents indicated that less than half of the respondents consumed enough fruit and vegetables and only a third exercised regularly. Analysis of adherence to treatment showed that more than half of the respondents who were prescribed daily medication did not take them for various reasons, mainly because of “Fear of side effects”, “Financial situation”, and “Lack of information about benefits of drug therapy”. These results may indicate that patients underestimate the importance of a healthy lifestyle and their prescribed treatment and do not realize that the benefits they offer outweigh the drawbacks of drug therapy and associated future medical costs.

The main reasons for non-adherence to healthy lifestyle as experienced and answered by all the respondents were “Lack of knowledge”, “Financial situation”, and “Lack of motivation” (**Tables 2**, **3**). It is interesting to note that the main reason indicated by most of the respondents without a diagnosis of diabetes was “Lack of knowledge”, while that indicated by most of the respondents with a diagnosis of diabetes was “Financial situation”. These differences might be explained by the fact that the respondents with diabetes mellitus were under the observation of health care professionals and received more information about the disease compared with the respondents without diabetes. One finding that highlights the positive effect of lifestyle advice from health care professionals was that alcohol consumption was lower among those with diabetes. These patients were more informed and motivated to adhere to the principles of a healthy lifestyle, but due to the substantially higher financial costs they faced in order to maintain their health, their top reason for non-adherence was “Financial situation”. Increasing out-of-pocket payments is one of the critical issues facing the health care system in Belarus (17). The proportion of those selecting “Financial situation” differed depending on the diagnosis of diabetes, gender, and education, which suggests further investigation would be warranted to determine whether there is a potential financial gap for accessing appropriate diabetes care.

The Diabetes Attitudes, Wishes, and Needs (DAWN) study was a cross-sectional international survey initiated in 2001 by Novo Nordisk in collaboration with the International Diabetes Federation. The purpose of the survey was “to identify a broad set of attitudes, wishes, and needs among both people with diabetes and care providers to lay a foundation for efforts to improve diabetes care nationally and internationally” (18). The DAWN study highlighted that diabetes can lead to numerous psychosocial issues, and that such issues are themselves obstacles to self-management, negatively impacting on the patient’s ability to achieve sufficient glycemic control. The study also noted that existing health care systems are inadequately designed and equipped for dealing with chronic illnesses such as diabetes (18). These findings reflected the long-held views of both health care professionals and those affected by the illness. As practical actions to be taken, the study listed awareness raising and advocacy, education of patients and those at risk, and training of health care providers (18). Given that a lack of motivation for adhering to healthy lifestyle advice was highest among the youngest group in our study, more advocacy and education efforts for prevention and self-management of diabetes should be directed towards this younger generation, so that further increases in incidence rates and related complications can be prevented. In addition, insufficient knowledge was voiced more frequently among those with a lower educational attainment. Information development and provision should be attentive to the health literacy level of the target audience.

Regarding survey respondents’ perceived barriers that impede adherence to a healthy lifestyle among the general population in Belarus, the most common responses were “Financial situation”, “Lack of knowledge”, and “Lack of motivation” (**Table 4**), which were the same key barriers for patients themselves not following lifestyle instructions. Similarly, a higher proportion of those with diabetes selected “Financial situation”. On the other hand, those without diabetes selected “Lack of motivation” more frequently. There is a direct relationship between awareness of risk factors and motivation to make lifestyle changes. A qualitative study has shown that providing prediabetic adults with specific information about their risk of developing diabetes and the expected reduction in risk associated with preventive treatments might encourage lifestyle changes (19). Awareness on diabetes prevention and subsequent lifestyle changes among both patients and the general public can be facilitated by incorporating patient/resident-centered dialogues with detailed explanation about the benefits of preventive measures.

In conclusion, lack of knowledge about risk factors for diabetes in the general population and lack of adherence to preventive measures and treatment are persistent and important problems in the present health care system in Belarus. The findings of this study highlight the importance of taking a gradual approach to improving adherence to preventive measures and treatment for diabetes. Initially, it will be necessary to achieve an understanding among the general population of the role of risk factors in the development of the disease and the importance and benefits of drug therapy in patients. At the same time, through an investigation focusing on patients’ perspectives such as ours, medical care providers are expected to better recognize the difficulties that their patients face in preventing and treating diabetes, and better understand the benefits of positively seeking patient input during the consultation to achieve improved adherence. One of the co-authors is implementing a university–medical association collaborative initiative promoting skills among physicians in Vietnam that include listening to patients and analyzing text data (20). Such a trial might also be beneficial in Belarus.

### Limitations of the Study

The major methodological shortcoming of our study was its cross-sectional and descriptive nature with limited data on patients’ backgrounds, which did not allow us to run multivariate analysis and make causal inferences. In addition, the data collected were only quantitative, with given answer options; for example, respondents could not expand on the in-depth reasons behind the answer option “Other”, and self-reporting consumption of 400 grams of fruit and vegetables per day depended on robust estimates by the respondents. More detailed data collection including qualitative data in further research would help us more accurately assess patients’ health behaviors and better understand the difficulties that they experience in complying with healthy lifestyle instructions and treatment regimens.

## Data Availability Statement

The raw data supporting the conclusions of this article will be made available by the authors, without undue reservation.

## Ethics Statement

The studies involving human participants were reviewed and approved by Local executive committee of Gomel State Medical University. The patients/participants provided their written informed consent to participate in this study.

## Author Contributions

AS, TS, DK, MR, and IS contributed to designing the study, planning the data collection procedures, and collecting data. JT translated related documents including questionnaires. DK and AS analyzed data, and all members participated in interpretation of obtained results. AS wrote the first draft, and AG, HY, and AK took part in revision of the paper. All authors contributed to the article and approved the submitted version.

## Funding

The research was supported by the Program for the Network-Type Joint Usage/Research Center for Radiation Disaster Medical Science 2020 and 2021.

## Conflict of Interest

The authors declare that the research was conducted in the absence of any commercial or financial relationships that could be construed as a potential conflict of interest.

## Publisher’s Note

All claims expressed in this article are solely those of the authors and do not necessarily represent those of their affiliated organizations, or those of the publisher, the editors and the reviewers. Any product that may be evaluated in this article, or claim that may be made by its manufacturer, is not guaranteed or endorsed by the publisher.
